# Survey of endocrinologists managing recovery from anabolic androgenic steroid induced hypogonadism

**DOI:** 10.1530/RAF-22-0097

**Published:** 2023-02-27

**Authors:** Bonnie Grant, Anjali Pradeep, Suks Minhas, Waljit S Dhillo, Richard Quinton, Channa N Jayasena

**Affiliations:** 1 Department of Endocrinology and Metabolism, Barts Health NHS Trust, West Smithfield, London; 2 Section of Investigative Medicine, Imperial College London, Commonwealth Building, Hammersmith Hospital, Du Cane Road, London; 3 Department of Urology, Charing Cross Hospital, Fulham Palace Road, London; 4 Department of Endocrinology, Diabetes & Metabolism, Newcastle-upon-Tyne Hospitals NHS Foundation Trust & Translational & Clinical Research Institute, University of Newcastle-upon-Tyne, UK

**Keywords:** anabolic-androgenic steroids, hypogonadism, testosterone, withdrawal

## Abstract

Anabolic steroids (also known as ‘steroids’) are banned drugs like testosterone, which make muscles bigger in men. These drugs are dangerous because they stop the testes from making natural testosterone and can cause heart attacks. Men stopping steroids have very low testosterone, which makes them feel weak, depressed, suicidal, infertile, and unable to have erections. We surveyed over 100 doctors to find out how they treat men giving up steroids. We report that doctors differ widely in the way they treat these men. Most doctors simply advise men to wait for the natural recovery of testosterone levels to happen. But 20% of doctors give men drugs to boost testosterone and make men feel better. Unfortunately, many patients had not recovered by the time of our survey. In summary, our survey highlights differences and limitations in the treatment of men giving up steroids. The use of steroids is increasing rapidly among young men, so we recommend further work to improve the treatment of men who are motivated to give up steroids.

Anabolic-androgenic steroids (AAS) are a diverse range of drugs including testosterone derivates, gonadotrophins such as human chorionic gonadotrophin, and selective oestrogen receptor modulators (SERMs), which have direct or indirect androgen actions in the body. AAS are abused by men to artificially increase physical strength and muscular development (Anawalt 2019, Mullen *et al.* 2020). The lifetime prevalence of AAS use in men is estimated to be 1–5% worldwide (Anawalt 2019). Furthermore, the UK National Crime Survey of England and Wales estimated over 400,000 men used AAS, which represents an approximate doubling over a 10-year period (Mullen *et al.* 2020).

AAS profoundly suppress endogenous testosterone secretion through negative feedback on gonadotrophin-releasing hormone secretion. AAS cessation causes hypogonadism, often persisting for months to years (Kanayama *et al.* 2015, Shankara-Narayana *et al.* 2020), and commonly causes sexual dysfunction, infertility, depression, and suicidal ideation. Men with AAS-induced hypogonadism are routinely referred to hospital Endocrinology clinics. However, there are currently no management guidelines or recommended therapies for men with AAS-induced hypogonadism.

The clinical management of AAS-induced hypogonadism by clinicians has been under-reported. To address this, we conducted a voluntary, anonymous survey among attendees at the 2022 Endocrine Academy Clinical Update, organised by the Society for Endocrinology. Attendees included trainees (48%), consultants (16%), endocrine nurses (23%), and ‘other’ (13%). Questions covered experience, patient symptoms, patient outcomes, and confidence in managing men with AAS use (Supplemental Table 1, see section on supplementary materials given at the end of this article).

In total, 101 delegates responded to the survey of which 81 (80.2%) had experience reviewing someone recently stopping AAS. Clinicians had most commonly (30%) seen men within 3 months of AAS cessation (Fig. 1A). Frequently reported symptoms were reduced sex drive (77.8%), tiredness (70.4%), low mood (63%), and physical weakness (51.1%).

**Figure 1:**
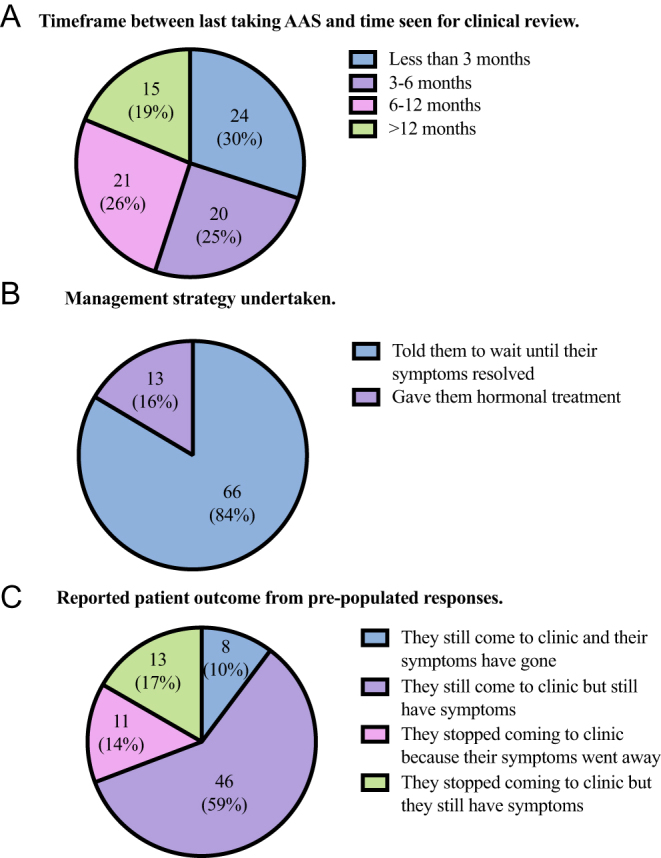
Clinician responses about the management of anabolic androgenic steroid withdrawal. (A) Timeframe between last taking AAS and time seen for clinical review. Number of respondents and percentage, *n* (%). Total *n* = 80, No response *n* = 1. (B) Management strategy undertaken. Number of respondents and percentage, *n* (%). Total *n* = 79, No response *n* = 2. (C) Reported patient outcome from pre-populated responses. Number of respondents and percentage, *n* (%). Total *n* = 78, No response *n* = 3.

Most (84%) responding clinicians advised men with AAS-induced hypogonadism to wait until symptoms resolved without any other treatment (Fig. 1B). The remaining 13 (16%) respondents provided hormonal treatment for symptoms. Most respondents (46/78; 59%) stated that their patient was still attending the clinic and experiencing symptoms of hypogonadal symptoms, as shown in Fig. 1C. Only a minority of respondents (18/92, 20%) were confident or extremely confident treating AAS-induced hypogonadism.

AAS use is rising rapidly within the young male population, so endocrinologists may expect increasing numbers of referrals for managing AAS-induced hypogonadism. Our survey is limited by potential recall bias, but reassuringly reports symptoms of AAS in agreement with prior research (Kanayama *et al.* 2015, Anawalt 2019). Importantly, we highlight divergence among clinicians in treating AAS-induced hypogonadism, either conservatively or with hormonal treatment (such as testosterone or SERM). It is also unsurprising that few endocrinologists were confident about treating AAS-induced hypogonadism, partly explained by the perception that their knowledge about AAS is inferior to fitness coaches, bodybuilding websites, and other AAS users (Bonnecaze *et al.* 2020). We cannot exclude differences in knowledge between consultant endocrinologists compared with trainees. We suggest further studies aim to define AAS-induced hypogonadism as a separate entity. This would support the development of evidence-based treatments and clinical guidance to optimise reproductive recovery in men motivated to stop AAS.

## Supplementary Material

Supplemental Table 1: Content of online study survey to endocrinology clinicians.

## Declaration of interest

R Quinton is an Associate Editor of *Reproduction and Fertility*. R Quniton was not involved in the review or editorial process for this paper, on which he is listed as an author.

## Funding

NIHR Post-Doctoral fellowship: C N Jayasena;, NIHR Senior Investigator: W S Dhillo.

## Author contribution statement

BG, CNJ, and RQ conceptualised the study and collected data. AP and BG did data analysis. BG, AP, and CNJ drafted the manuscript. WSD, RQ, and SM edited the manuscript. All authors approved the final submitted version.
