# Supplementary material for: Survey of endocrinologists managing recovery from anabolic androgenic steroid induced hypogonadism
Source: Reprod Fertil. 2023 Feb 27;4(1):e220097. doi: 10.1530/RAF-22-0097 (PMC9986377; doi:10.1530/RAF-22-0097)
Supplement: Supplemental Table 1: Content of online study survey to endocrinology clinicians. [file supplementary_table_1.pdf]

Supplemental Table 1: Content of online study survey to endocrinology clinicians.

1. Have you ever seen someone who recently stopped taking anabolic steroids?<br>• Yes<br>• No
2. What symptoms did they complain of? (Tick all that apply)<br>• I don't remember<br>• They had no symptoms<br>• Cravings to restart anabolic steroid use<br>• Low mood<br>• Anxiety<br>• Reduced sex drive<br>• Tiredness<br>• Suicidal thoughts<br>• Headaches<br>• Physical weakness<br>• Other
3. How did you manage them?<br>• Told them to wait until their symptoms recovered<br>• Gave them hormonal treatment<br>• Other
4. When had they last taken anabolic steroids?<br>• Less than 3 months<br>• 3-6 months<br>• 6-12 months<br>• Over 12 months
5. What happened to your patient?<br>• They still come to clinic and their symptoms have gone<br>• They still come to clinic, but they still have symptoms<br>• They stopped coming to clinic because their symptoms went away<br>• They stopped coming to clinic, but they still had symptoms
6. How confident are you treating men stopping anabolic steroids?<br>• 1-5 (Not confident at all to extremely confident).
